# Determination of anti-leishmanial drugs efficacy against *Leishmania martiniquensis* using a colorimetric assay

**DOI:** 10.1016/j.parepi.2020.e00143

**Published:** 2020-02-19

**Authors:** Atchara Phumee, Narissara Jariyapan, Saranyou Chusri, Thanaporn Hortiwakul, Oussama Mouri, Frederick Gay, Wacharee Limpanasithikul, Padet Siriyasatien

**Affiliations:** a Vector Biology and Vector Borne Disease Research Unit, Department of Parasitology, Faculty of Medicine, Chulalongkorn University, Bangkok, Thailand; b Department of Parasitology, Faculty of Medicine, Chiang Mai University, Chiang Mai, Thailand; c Division of Infectious Diseases, Department of Internal Medicine, Faculty of Medicine, Prince of Songkla University, Songkhla, Thailand; d Sorbonne Universite - Faculty of Medicine, AP-HP - Groupe Hospitalier Pitié-Salpêtrière, Paris, France; e Department of Pharmacology, Faculty of Medicine, Chulalongkorn University, Bangkok, Thailand

**Keywords:** *Leishmania martiniquensis*, Drug susceptibility, Colorimetric assay, *In vitro*, *Ex vivo*

## Abstract

Autochthonous leishmaniasis caused by *Leishmania martiniquensis* cases in Thailand have dramatically increased in the recent years. *L. martiniquensis* infection primarily occurs in immunocompromised patients, especially AIDS patients. In Thailand, amphotericin B is the only drug available for leishmaniasis treatment, and some patients relapse after amphotericin B therapy. Moreover, the efficacy of anti-leishmanial drugs against *L*. *martiniquensis* has not been evaluated to date. In this study, we determined the efficacy of various anti-leishmanial drugs against the promastigote and intracellular amastigote stages of *L*. *martiniquensis* using a colorimetric assay. Two strains (CU1 and CU1R1) were isolated from leishmaniasis HIV co-infected patient from Songkhla province, southern Thailand. The CU1 strain was isolated from the patient in 2011, and CU1R1 was isolated from the same patient in 2013, when he was diagnosed as relapse leishmaniasis. The third strain (LSCM1) used in this study has been isolated from immunocompetent patient from Lamphun province, northern Thailand. All strains were identified as *L*. *martiniquensis* by sequencing of ribosomal RNA ITS-1 and large subunit of RNA polymerase II gene. Bioassays have been conducted both with promastigote and intracellular amastigote stages of the parasite. All *L*. *martiniquensis* strains have been tested against amphotericin B, miltefosine and pentamidine to determine the efficacy of the drugs against the parasite by using a PrestoBlue. The efficacy of miltefosine and pentamidine exhibit no significant difference between each stage of *L*. *martiniquensis* among all strains. Surprisingly, the promastigote and intracellular amastigote of the CU1R1 isolate, which was isolated from a relapsed patient after amphotericin B treatment, exhibited a two-fold increased inhibitory concentration (IC50) against amphotericin B compared with other strains, and the difference was statistically significant (*p* < 0.05). Moreover, intracellular amastigotes isolated from CU1R1 exhibited slightly increased susceptibility to amphotericin B compared with the promastigote (*p* < 0.05). The result of this experiment is a scientific evident to support that in case of relapsed leishmaniasis caused by *L*. *martiniquensis*, increasing dosage of amphotericin B is essential. Moreover, this study also determined efficacy of other anti-leishmanial drugs for treatment the leishmaniasis in Thailand in case of these drugs are available in the country and the clinicians should have alternative drugs for treatment leishmaniasis in Thailand apart from amphotericin B.

## 1 Introduction

*Leishmania* is protozoa belonging to the Family *Trypanosomatidae* of the Order *Kinetoplastida* (Lainson and Shaw, 1987). These protozoans are parasites responsible for leishmaniasis. The parasites are transmitted to vertebrate hosts by the bite of female sand flies (Bates, 2007). The parasites have dimorphic life cycle, including an intracellular amastigote form and a promastigote form. The amastigote is characterized by a round or ovoid shape. The immotile amastigote multiplies in the cytoplasm of macrophages of vertebrate hosts. The promastigote form is characterized by a spindle-shape. This form uses a flagellum for motility and is found in female sand fly (Bates, 2007; Kato et al., 2010). Leishmaniasis occurs in tropical and subtropical areas and southern Europe (Steverding, 2017). Leishmaniasis is classified into the Old World and New World leishmaniasis. Old World leishmaniasis is caused by the subgenus *Leishmania*, which is found in Africa, Asia, the Middle East, the Mediterranean, and India. New World leishmaniasis is found in Central and South America and is caused by the *L*. *mexicana* complex and all species of the subgenus *Viannia* (Maltezou, 2010). There are three main clinical forms of the disease, including visceral, cutaneous, and mucocutaneous leishmaniasis (Lainson and Shaw, 1987; Maltezou, 2010). Clinical forms and treatment depend on the *Leishmania* species (Mouri et al., 2014). In Thailand, indigenous leishmaniasis is an emerging disease described in recent years. The disease was reported in northern, central and southern regions of Thailand (Maharom et al., 2008). Two major *Leishmania* species *L*. *martiniquensis* and *L*. *siamensis (L. orientalis n. sp.)* (Sukmee et al., 2008; Suankratay et al., 2010; Bualert et al., 2012; Chusri et al., 2012; Phumee et al., 2013; Noppakun et al., 2014; Osatakul et al., 2014; Phumee et al., 2014; Pothirat et al., 2014; Chiewchanvit et al., 2015; Siriyasatien et al., 2016; Jariyapan et al., 2018) were reported in Thailand. Several drugs are effective for the treatment of leishmaniasis, including pentavalent antimonials, amphotericin B deoxycholate, lipid formulations of amphotericin B, miltefosine, paromomycin, pentamidine and sodium stibogluconate; however, these drugs exhibit different modes of action, adverse effects, costs, and routes of administration (Croft and Brazil, 1982; Singh and Sivakumar, 2004; Singh, 2006; Morizot et al., 2016). Currently, several reports described the increasing drug resistance in many *Leishmania* species (Croft et al., 2006; Mohapatra, 2014). However, the level of resistance varies according to the *Leishmania* species, strain, stages of *Leishmania* and the distribution and frequency of resistance to anti-leishmanial drugs are unknown. The variation in intrinsic sensitivity among some *Leishmania* species to several drugs depends on genetic, molecular, and biochemical variations. In Thailand, amphotericin B is the only effective drug available for the treatment of leishmaniasis; however, some patients developed relapsed leishmaniasis after receiving amphotericin B treatment (Suankratay et al., 2010; Chusri et al., 2012; Phumee et al., 2013; Phumee et al., 2014; Pothirat et al., 2014; Siriyasatien et al., 2016). Moreover, the efficacy of amphotericin B and other anti-leishmanial drugs against *L*. *martiniquensis* has never been evaluated. Therefore, in this study, we sought to determine the efficacy of anti-leishmanial drugs against various strains of *L*. *martiniquensis* isolated from Thai patients using colorimetric assays. The resazurin-based assay PrestoBlue™ Cell Viability Reagent was used because it is a non-toxic, water soluble, redox-sensitive dye. Data obtained from this study may potentially improve the treatment of leishmaniasis caused by *L*. *martiniquensis* in Thailand.

## 2 Material and methods

### 2.1 Promastigote of *Leishmania martiniquensis* strains

Three *Leishmania martiniquensis* strains were used for drug sensitivity tests, including CU1, which was isolated from an HIV-*Leishmania* co-infected patient from Songkhla province, southern Thailand. The culture originated from bone marrow aspirate. The patient was treated with intravenous amphotericin B deoxycholate (Chusri et al., 2012; Phumee et al., 2013). The second isolate is CU1R1, which was isolated from the same patient. The patient developed cutaneous nodules 2 years after the initial amphotericin B treatment (Phumee et al., 2014). The final isolate is LSCM1, which was isolated from the bone marrow of a Thai immunocompetent patient from Lamphun province, northern Thailand. The isolate of this strain was assigned WHO code MHOM/MQ/92/MAR1; LEM2494. The patient was treated with intravenous amphotericin B deoxycholate (Pothirat et al., 2014). 50–100 μl of bone marrow aspirate was loaded into 5 ml Schneider's insect medium (Sigma-Aldrich, USA) containing 10% fetal bovine serum and 100 U/100 μg/ml penicillin-streptomycin (Sigma-Aldrich, USA) in a 25-cm^3^ flask and maintained at 25 ± 2 °C and were inspected for parasites every 24 h under an inverted microscope (Olympus, Japan). For continuous maintenance, the cultures were passaged every 2–3 days by diluting the original culture with Schneider's media at 1:2 dilutions.

### 2.2 Macrophage cell culture

Murine macrophages J774A.1 cells were purchased from American Type Culture Collection and were cultured in Dulbecco's modified Eagle's medium (DMEM) supplemented with 10% heat-inactivated fetal bovine serum at 37 °C in 5% CO_2_ in a humidified atmosphere. The cultures were inspected for the parasites every 24 h under an inverted microscope (Olympus, Japan).

### 2.3 Determination of the number of *Leishmania* parasites and macrophage cells

Twenty microliters of promastigote cells or macrophage cells were obtained from 1 ml of cultures and gently mixed with 20 μl of trypan blue solution stock at a dilution factor of 2. Then, 10 μl of the stained cell mixtures was transferred onto the hemocytometer and incubated at room temperature for 5 min. Viable cells, which were unstained, were counted in 5 squares under a microscope at 400× magnification. The number of cells was calculated using the following equation:

$$\;\text{Number of cells}\;\left(\text{cells}/\mathrm{ml}\right)=\text{Average cells}\times 104\times \text{Dilution factor}$$

### 2.4 Infection of J774A.1 macrophage cells with *L*. *martiniquensis*

J774A.1 cells were infected with stationary phase promastigotes in a 24-well plate at a ratio of 10:1 (parasites/macrophage) or 10^6^:10^5^ cells/ml/well and incubated at 37 °C in 5% CO_2_ for 24 h. The cells were washed with DMEM medium to remove non-phagocytosed parasites. Twenty-four hours after the experiment, *Leishmania* infections were confirm by structural changes in host cells by Giemsa staining. The intracellular amastigotes was demonstrated using an inverted, light microscope (Olympus, Japan).

### 2.5 Anti-leishmanial agents

The promastigotes of *L*. *martiniquensis* (1 × 10^6^ cells/ml/well) were transferred into 96-well culture plates or intracellular amastigotes (1 × 10^6^ cells/ml/well) in 96-well culture plates. Both of stages were exposed to different concentrations of anti-leishmanial drugs. The experiments were performed in triplicate amphotericin B from *Streptomyces* sp. (Sigma-Aldrich, USA) was used at concentrations of 2, 1.5, 1, 0.5, 0.3, 0.25, 0.2, and 0.15 μM miltefosine (Sigma-Aldrich, USA) was used at concentrations of 60, 50, 40, 30, 20, 15, 10, and 5 μM. Pentamidine (Sigma-Aldrich, USA) was used at concentrations of 60, 50, 30, 20, 10, 8, 5, and 3 μM. Cultures treated with diluents without anti-*Leishmania* agents were used as controls. Seventy-two hours after the treatment, living cells were quantified, and IC50 values were calculated.

### 2.6 Colorimetric assay of living cells

PrestoBlue™ is a resazurin-based solution reagent that functions as a cell viability indicator, utilizing the reducing power of living cells to quantitatively measure the proliferation of cells. The PrestoBlue™ reagent is a cell-permeable compound that is blue in color and virtually non-fluorescent. When added to cells, PrestoBlue™ is modified by the reducing environment of the viable cell and becomes red and highly fluorescent. The method was performed as follow. First, 90 μl of 1 × 10^6^ cells/ml in vitro culture of promastigotes or ex vivo intracellular amastigotes were added into 96-well plates. In addition, blank wells containing only culture media (no cells) were included on each plate. Anti-leishmanial compounds, including amphotericin B, miltefosine, and pentamidine, were used at different concentrations as described previously. Seventy-two hours after incubation with anti-leishmanial drugs, 10 μl of PrestoBlue was added directly into existing hanging drops containing the samples and control media. The samples were incubated for an additional 60 min at 37 °C. Inhibition was determined by using a fluorescence plate reader at 560 nm excitation and 590 nm emission, and the percent of inhibition was calculated using the formula presented below:

$$\%\text{Inhibition}=\frac{\text{Absorbance}\;\left(\text{control}\right)-\text{Absorbance}\;\left(\text{sample}\right)\;}{\text{Absorbance}\;\left(\text{control}\right)}\times 100$$

The percent inhibition of different drug concentrations and calculations for the IC50 value were performed using GraphPad Prism version 6 program (San Diego, USA).

### 2.7 Data analysis

IC 50 value of promastigotes and intracellular amastigotes were calculated for the mean and standard deviation (SD) from triplicate experiments. Statistical analysis was performed using the statistical package for social sciences (SPSS) software, version 16.0 for Windows (SPSS Inc., Chicago, USA). The correlations between drugs and isolations were determined using one-way ANOVA. *p* < 0.05 was considered to be statistically significant for differences and correlations. Differences in promastigote and intracellular amastigote forms for each drug were compared for statistical analysis using an independent *t*-test. A *p*-value < 0.05 was regarded as indicating statistical significance. The IC_50_ values of each anti-leishmanial drug and each *Leishmania* stage are presented in the table.

## 3 Results

### 3.1 *In vitro* screening assay

**Fig. 1:**
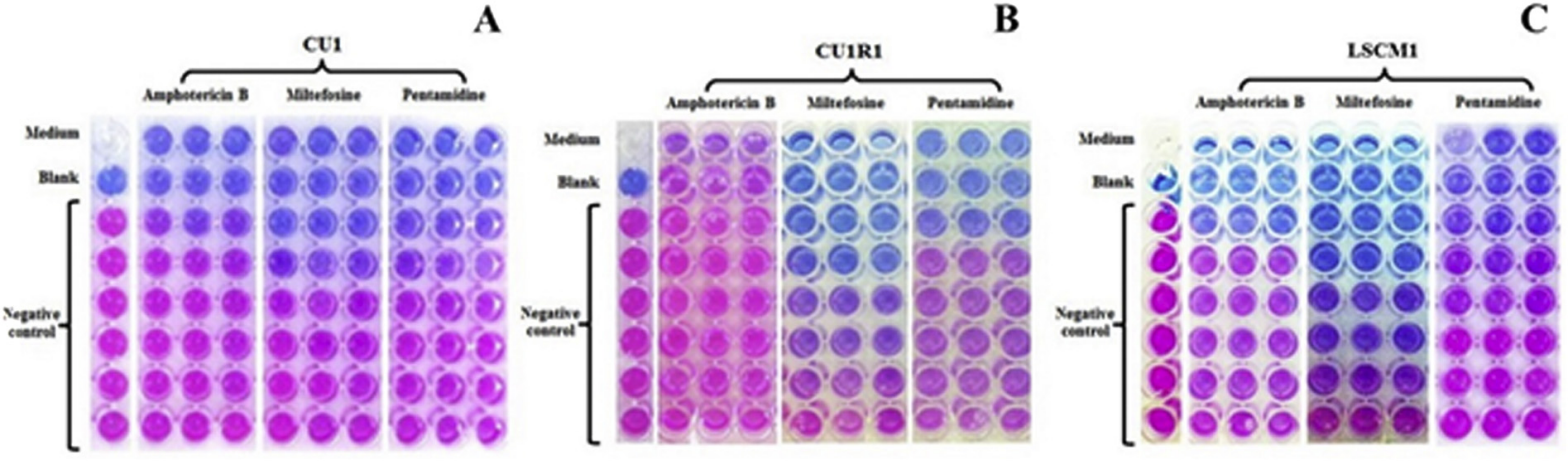
Colorimetric assay using the PrestoBlue reagent; amphotericin B, miltefosine and pentamidine were test against three *L*. *martiniquensis* promastigote strains CU1, CU1R1 and LSCM1 as shown in panels A, B and C, respectively.

**Fig. 2:**
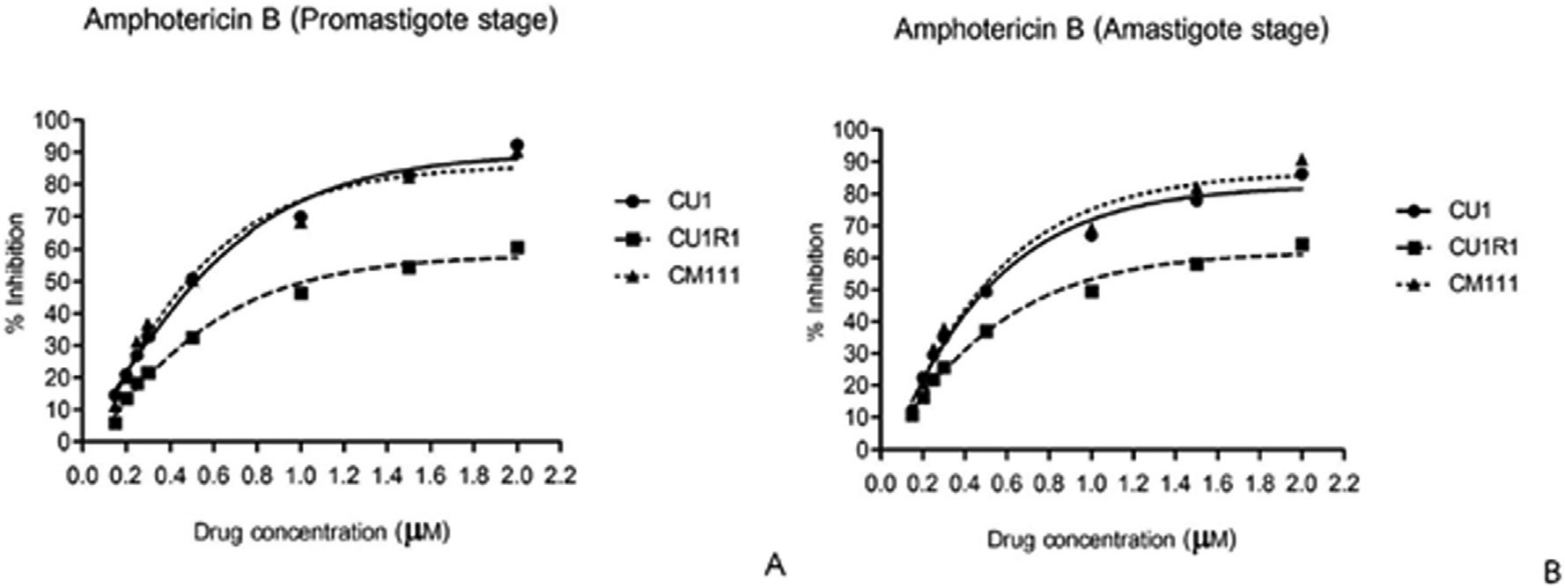
Comparative of % inhibition in promastigote (A) and amastigote (B) stages with amphotericin B. Experiments were performed in triplicate.

*In vitro* means ‘in glass’, the experiment were conducted outside organisms, in this study is *in vitro* was refer to promastigote form. IC50 values were calculated from % inhibition values of anti-leishmanial drugs against the parasites, which was determined by the change in the color of PrestoBlue (Fig. 1). IC50s of amphotericin B were 0.497 ± 0.128 μM, 1.025 ± 0.065 μM, and 0.475 ± 0.08 μM for CU1, CU1R1 and LSCM1, respectively (Table 1). IC50 value of the CU1R1 isolate was two-fold increase compared with other isolations (*p*-value < 0.05) (Fig. 2), whereas the efficacy of amphotericin B against CU1 and LSCM1 promastigotes was similar. The mean IC50 values of miltefosine were 17 ± 0.173 μM, 17.067 ± 0.065 μM, and 18.4 ± 0.08 μM for CU1, CU1R1 and LSCM1, respectively. The LSCM1 isolate exhibited the lowest susceptibility to miltefosine compared with CU1 and CU1R1. The IC50 of pentamidine were 12.967 ± 0.289 μM, 13.967 ± 0.404 μM, and 13.133 ± 0.289 μM against CU1, CU1R1 and LSCM1, respectively. The CU1R1 isolate exhibited significantly reduced susceptibility compared with CU1 and LSCM1.

**Table 1:** Determination of IC50 values of anti-leishmanial drugs in promastigote and intracellular amastigote stages using the colorimetric prestoblue rezasurin-based assay.

Drug name	IC50 value ($\bar{X}\pm \mathrm{SD}$) (μM)					
	Promastigote form			Intracellular amastigote form		
	CU1	CU1R1	LSCM1	CU1	CU1R1	LSCM1
amphotericin B	0.497 ± 0.127	1.025 ± 0.065⁎	0.475 ± 0.08	0.483 ± 0.217	0.856 ± 0.172⁎	0.486 ± 0.207
miltefosine	17 ± 0.173	17.067 ± 0.252	18.4 ± 0.8	18.933 ± 0.737	19.267 ± 0.404	17.433 ± 0.416
pentamidine	12.967 ± 0.289	13.967 ± 0.404⁎	13.133 ± 0.289	12 ± 0.7	12.8 ± 1.1	12.7 ± 0.346

⁎ Significant difference (*p*-value < 0.05).

### 3.2 *Ex vivo* screening assay

*Ex vivo* means ‘out of living’, it refers to experiment done in or on living tissue in an artificial environment outside the organism, in this study is refer to intracellular amastigote form. The efficacy of amphotericin B against CU1, CU1R1, and LSCM1 as indicated by IC50 values was 0.483 ± 0.217 μM, 0.856 ± 0.172 μM, and 0.486 ± 0.207 μM, respectively. Interestingly, the IC50 of the CU1R1 isolate exhibited a significant two-fold increase compared with other strains (*p*-value < 0.05), whereas no significant difference were noted between CU1 and LSCM1. The IC50 of miltefosine were 18.933 ± 0.737 μM, 19.267 ± 0.404 μM, and 17.433 ± 0.416 μM for CU1, CU1R1 and LSCM1, respectively. The LSCM1 isolate exhibited increased efficiency compared with CU1 and CU1R1 strains. However, mean IC50 against CU1 and CU1R1 were not significantly different. The IC50 of pentamidine were 12.0 ± 0.7 μM, 12.8 ± 1.1 μM, and 12.7 ± 0.346 μM for CU1, CU1R1 and LSCM1, respectively. The susceptibility of all strains exhibited no significant differences. The efficacy of each anti-leishmanial agent (amphotericin B, miltefosine, and pentamidine) was determined against the intracellular amastigote and promastigote forms. The IC50s of each drug were summarized in Table 1. The intracellular amastigote stage was slightly more susceptible to amphotericin B compared with the promastigote stage. A similar trend was observed for miltefosine and pentamidine with comparable efficacies against the promastigote and intracellular amastigote forms.

## 4 Discussion

*Leishmania martiniquensis* infection is an emerging disease in Thailand (Pothirat et al., 2014; Chiewchanvit et al., 2015) and >90% of indigenous leishmaniasis cases in the country are caused by *L*. *martiniquensis*. In Thailand, a limited number of anti-leishmanial agents are available for treatment of *L*. *martiniquensis* infection. Amphotericin B is the only drug available for the treatment of leishmaniasis in Thailand. Moreover, the efficacy of anti-leishmanial agents against *L*. *martiniquensis* has not been determined to date. Current approaches for anti-leishmanial screening include quantitative microscopy, radioactive nucleotides, colorimetric assays, and reporter gene-based assays. The reporter gene can be used for rapid and high-throughput drug screening systems utilizing green fluorescent protein (GFP), luciferase, and β-galactosidase (Bastos et al., 2017; Bolhassani et al., 2011; Pulido et al., 2012). Several colorimetric assays have been described to determine the viability of *Leishmania* parasites, such as measuring enzyme activities and cleavage of the tetrazolium salt 3-(4,5-dimethyl-thiazol-2-yl)-2,5-diphenyl-tetrazolium bromide (MTT) (Ganguly et al., 2006; van den Bogaart et al., 2014; Xu et al., 2015). However, all these methods require subsequent manipulation steps and are time consuming and toxic to the cell (Callahan et al., 1997). In this study, we also demonstrated using PrestoBlue to determine the anti-leishmanial effects of various compounds against *L*. *martiniquensis*. This technique is simple, requires only basic equipment, is easy to implement and uses a one-step assay to measure drug activity against *L*. *martiniquensis* intracellular amastigotes grown in J774A.1 cells and promastigotes with the rezasurin-based assay. Resazurin (blue and non-fluorescent) is reduced to resorufin (pink and highly fluorescent). Erb and Ehlers (1950) reported that the resazurin reduction test has been used since the 1950s to monitor bacterial or yeast contamination of milk and assess semen quality (Erb and Ehlers, 1950; Carter et al., 1998). In 1997, Raz and others determined the *in vitro* drug sensitivity of African trypanosomes (*Trypanosoma brucei gambiense* and *T. b. rhodesiense*) using the Alamar Blue® rezasurin-based assay, which is a non-toxic cellular assay that can be used with long incubation periods (Raz et al., 1997). Mikus and Steverding (2000) measured the cytotoxicity of compounds against the promastigote of *L*. *major* using the oxidation-reduction indicator Alamar Blue® (Mikus and Steverding, 2000). The results revealed that the IC50 values of amphotericin B, pentostama (sodium stibogluconate), and paromomycin were 0.31 ± 0.07 μg/ml, 28.7 ± 2.0 μg/ml, and 50.6 ± 8.2 μg/ml, respectively. This assay was in the same range as previously determined by other methods. Nevertheless, the study of anti-leishmanial drug sensitivity using the PrestoBlue® rezasurin-based assay has not been determined for *L*. *martiniquensis* to date. Screening of anti-leishmanial drug for leishmanicidal activity can be performed with promastigotes, axenic amastigotes, and intracellular amastigotes, which are cultured under axenic conditions (Kloehn et al., 2015). The differences in environmental conditions between promastigotes and amastigotes in *in vivo* are reflected in their needs for *in vitro* cultivation. Amastigotes are more difficult to maintain *in vitro*, but promastigotes are easily cultured in media (Fumarola et al., 2004; Nolan and Bouchard, 1991). Several study results supported the intracellular amastigote model as the gold standard for *in vitro* of *Leishmania* drug discovery research. However, it remains unclear how these models cross-validate each other. Therefore, this study compared the IC50 values indicative of the drug sensitivity of promastigotes and intracellular amastigotes of 3 strains to 3 anti-leishmanial drugs (amphotericin B, miltefosine, and pentamidine). The IC50 results revealed that extracellular promastigotes and intracellular amastigotes of CU1R1 isolated from a relapsed patient after amphotericin B treatment exhibited two-fold increased IC50 values against amphotericin B compared with the other strains; moreover, the amastigote form (IC50, 0.856 ± 0.172 μM) exhibits slightly increased susceptibility to amphotericin B compared with the promastigote form (IC50, 1.025 ± 0.065 μM). No difference in susceptibility to miltefosine and pentamidine was observed for the intracellular amastigote and promastigote forms of the three strains. Information from clinicians stated that some patients were relapsed after received amphotericin B treatment (Phumee et al., 2014; Siriyasatien et al., 2016). The result of this experiment is a scientific evident to support that in case of relapsed leishmaniasis caused by *L*. *martiniquensis*, increasing dosage of amphotericin B is essential. This study revealed a similar trend of drug susceptibility compared to other *Leishmania* parasite reports. For example, Vermeersch et al. (2009) demonstrated that no significant different susceptibility of amphotericin B between axenic amastigotes and promastigote stages, but amphotericin B showed slightly higher potency against intracellular amastigotes (Vermeersch et al., 2009). The ED50 values of amphotericin B against *L*. *major* strain NEAL-P promastigotes and amastigote were 0.5 and 0.2 μg/ml, respectively. In addition, the ED50 values for the promastigote and amastigote forms of *L*. *major* strain JISH118 were 0.96 and 0.6 μg/ml, respectively (Yardley and Croft, 1997). In contrast, Callahan et al. (1997) found that the IC50s for *L*. *mexicana* promastigote and intracellular amastigote forms differed for pentostam, glucantime, paromomycin, and pentamidine by three-fold or greater (Callahan et al., 1997). The IC50s values in axenic amastigote and intracellular amastigotes differed by two-fold for only one drug. This study reported lower efficacies against the promastigotes and amastigotes of the CU1R1 isolate compared with CU1 and LSCM1 strains. The decreased susceptibility to amphotericin B was attributed to the fact that the CU1R1 isolate was obtained from a relapsed patient after amphotericin B treatment. Mbongo et al. (1998) investigated the development and mechanism of amphotericin B-resistant *L*. *donovani* promastigotes upon increasing drug exposure (Mbongo et al., 1998). The IC50 values of resistant cells were 20-fold increase compared with wild-type cells, which were stable after >40 passages in drug-free medium. Moreover, saturated fatty acids were prevalent in resistant cells. Stearic acid was the major fatty acid, and the major sterol was an ergosterol precursor, namely, cholesta-5, 7, 24-trien-3b-ol and not ergosterol as noted in the amphotericin B-sensitive strain. However, mechanisms of natural resistance are unclear. Sundar et al. (2000) revealed that in Bihar, India, 65% of untreated patients fail to respond promptly or relapse after therapy with antimony drugs due to the development of drug resistance (Sundar et al., 2000). The IC50 values of miltefosine in these three strains were similar to those in *L*. *infantum* strain MON29 (IC50, 15.5 μM), *L. donovani* strain AG83 (IC50, 17.5 μM), *L. tropica*, K27 (IC50, 18 μM), and *L*. *braziliensis* strain L280 (IC50, 17.5 μM) in all stages of *Leishmania* infections as assessed using an MTS assay based on the bio-reduction of the tetrazolium salt MTS (3-(4,5-dimethylthiazol-2-yl)-5-(3-carboxymethoxyphenyl)-2-(4-sulfophenyl)-2H tetrazolium, inner salt) into soluble formazan by mitochondrial dehydrogenase enzymes (Ganguly et al., 2006). The leishmanicidal activity of pentamidine in *L*. *martiniquensis* exhibits a higher IC50 compared with promastigotes of other species, such as *L*. *donovani* (IC50, 1.3 μM), *L. infantum* (IC50, 2.87 μM), *L. tropica* (IC50, 1.04 μM), *L. braziliensis* (IC50, 1.23 μM), *L. mexicana* (IC50, 2.61 μM), *L. amazonensis* (IC50, 1.32 μM), and *L*. *major* (IC50, 2.83 μM) as assessed with the MTS assay (Ganguly et al., 2006). Based on data on the response of sensitive parasites to anti-leishmanial drugs in each stage of *Leishmania* is used in random primary screens. Many reports suggested that the anti-leishmanial drugs affect intracellular but not axenic parasites, it depended on a species of *Leishmania* and a host cell-dependent mechanism of action (Callahan et al., 1997). However, differences in the drug susceptibilities of the two stages have not been addressed directly.

## 5 Conclusions

The PrestoBlue® Rezasusin-based assay was to evaluate drug activity against *L. martiniquensis*. This assay could be applied for preliminary screening of new anti-leishmanial agents and as an alternative tool in *Leishmania* research in the future. This report showed that IC50 against amphotericin B was two-fold increased for *L*. *martiniquensis* in the CU1R1 isolate from a relapsed patient compared with the other strains. Other antileishmanial drugs may be considered for use in Thailand. This study showed that miltefosine and pentamidine are effective *in vitro* and *ex vivo*. However, the efficacy of other drugs against *L*. *martiniquensis* should be determined in the future. This study also determined efficacy of other anti-leishmanial drugs for treatment the leishmaniasis in Thailand in case of these drugs are available and the clinicians should have alternative anti-leishmanial drugs apart from amphotericin B. Data presented in this study are valuable to improve treatment, reveal drug efficacy, and control drug-resistant *L*. *martiniquensis* in Thailand.

## Abbreviations

CU1: Promastigote was isolated from the patient in 2011
CU1R1: Promastigote was isolated from the same patient (CU1) in 2013, when he was diagnosed as relapse leishmaniasis.
LSCM1: Promastigote was isolated from immunocompetent patient.
In vitro: ‘in glass’, the experiment were conducted outside organisms, in this study *in vitro* was refer to promastigote form.
Ex vivo: ‘out of living’, it refers to experiment done in or on living tissue or cell line in an artificial environment outside the organism, in this study refer to intracellular amastigote form.
